# Protective effects of (1-(4-hydroxy-phenyl)-3-m-tolyl-propenone chalcone in indomethacin-induced gastric erosive damage in rats

**DOI:** 10.1186/s12917-014-0303-7

**Published:** 2014-12-31

**Authors:** Summaya M Dhiyaaldeen, Zahra A Amin, Pouya H Darvish, Iman Fahmi Mustafa, Mazen M Jamil, Elham Rouhollahi, Mahmood A Abdulla

**Affiliations:** Department of Biomedical Science, Faculty of Medicine, University of Malaya, 50603 Kuala Lumpur, Malaysia; Department of Pharmacognosy, College of Pharmacy, Hawler Medical University, 44001 Erbil, Iraq; Department of Microbiology, Faculty of Medicine, University of Malaya, 50603 Kuala Lumpur, Malaysia; Department of Molecular Medicine, Faculty of Medicine, University of Malaya, 50603 Kuala Lumpur, Malaysia; Center of studies for Periodontology, Faculty of Denistry, University Teknologi Mara, 40450 Shah Alam, Malaysia; Department of Pharmacology, Faculty of Medicine, University of Malaya, 50603 Kuala Lumpur, Malaysia

**Keywords:** Chalcone, Indomethacin, Peptic ulcer, Antioxidant, Immunohistochemistry, Histology, Endogenous enzymes

## Abstract

**Background:**

Non-steroidal anti-inflammatory drugs (NSAIDs) can result in peptic ulcer disease (PUD) which is a common condition worldwide. The aim of this study was to evaluate the antiulcer properties of (1-(4-hydroxy-phenyl)-3-m-tolyl-propenone) (HPTP) chalcone in rats using indomethacin as ulcerogenic agent.

**Results:**

None of the rats showed symptoms of kidney and liver toxicity during the term of the study. Administration of HPTP had decreased the acidity, increased gastric wall mucus and flattening of gastric mucosa and reducing erosive gastric damage area. HPTP also showed dose dependent increase in SOD, GPx activity and PGE_2_ level and decrease MDA. H & E stain showed decreased infiltration of leucocytes with edema of submucosal layer. PAS staining showed intense uptake of magenta color of gastric wall mucus in rats fed with HPTP, and immunohistochemical staining of gastric mucosa revealed over-expression of HSP70 protein, down-expression of Bax protein and over expression of TGF-β in rats administered with HPTP.

**Conclusion:**

This study has revealed that chalcone1-(4-hydroxy-phenyl)-3-m-tolyl-propenone can serve as a safe and effective antiulcer agent as it has been proved to increase pH and gastric wall mucus, increase GPx, SOD, PGE_2_, and decrease MDA level, ultimately, it has also contributes towards the over-expression of HSP protein andTGF-β, and down-expression of Bax protein.

**Electronic supplementary material:**

The online version of this article (doi:10.1186/s12917-014-0303-7) contains supplementary material, which is available to authorized users.

## Background

Peptic ulcer disease (PUD) is a common condition as contributes to morbidity and mortality in humans worldwide. The annual prevalence of PUD is 0.1-0.19% [1]. Aetiology of PUD is multi-factorial [2]. High acid secretion and decreased mucosal barrier contribute to initiation and progression of PUD [1]. The aetiology is ranging from *Helicobacter pylori* (*H-pylori*) infection to stress, lifestyle characteristics and drugs [3]. One of the most important drugs that contribute to PUD is non-steroidal anti-inflammatory drugs (NSAID) [4]. Consumption of NSAIDs is shown to be related to increased mucosal injury, erosions and gastric and duodenal bleeding. This effect is mainly due to the inhibition of the cyclooxygenase 1 (COX1) resulting in reduced mucosal defence as well as Thromboxane A_2_ which regulates platelet aggregation and therefore results in gastric bleeding [5]. Another mechanism that is well described in NSAIDs induced gastric injury is the production of reactive oxygen species (ROS) [6]. In literatures, several authors had been reported the use of synthesized compound for the treatment of gastrointestinal disorders including gastric ulcerogenic damage [7-9].

The main goals of PUD treatment, is firstly to reduce acid secretion, the hallmark of these medications is the proton pomp inhibitor (PPI), and secondly to improve mucosal barrier, including sucralfate and bismuth salts (cytoprotective agents) [10]. It is also believed that scavenging free radicals will also prevent the production of PUD [6]. After discovery of *H-pylori*, eradication of this bacterial infection became one of the essential steps in treatment of PUD [10,11]. Herbal medication and natural substances were long used for the treatment and management of PUD. Different herbs and spices have been introduced, in traditional medicines, as treatments for PUD. Plant derived medications have long been considered as safe and effective agents against PUD [12].

Flavonoids are one of the most abundant compartments in the vegetarian kingdom. Flavonoids are found in any part of plants including leaves, stalks, roots, fruits and seeds and are proven to have different effects on the gastrointestinal tract. They are also shown to be effective in prevention and treatment of PUD [13]. Chalcones are the precursors of flavonoids in plants. Most chalcones contain a six-member heterocyclic ring and are considered the first product of flavonoid synthesis pathway [14]. Various chalcones have been identified in nature and synthesized in laboratories and were shown to have variety of effects including prevention of peptic ulcer and oxidative stress as well as antibiotic, antifungal and anti-inflammatory effects [14-16]. Among the variety of chalcones 38 chalcones were shown to have antiulcer effects due to increased gastric blood flow and stimulating mucosal secretion. To the best of our knowledge no study was performed on the protective effect of chalcone against ulcers induced by NSAID prescription as well as the antioxidant properties of this chalcone in NSAID induced gastric injury.

The aim of this study was to identify the effects of chalcone (1-(4-hydroxy-phenyl)-3-m-tolyl-propenone**)** (HPTP) on NSAID induced erosive gastic damage in *Sprague Dawley* rats.

## Results

### Acute toxicity

**Figure 1:**
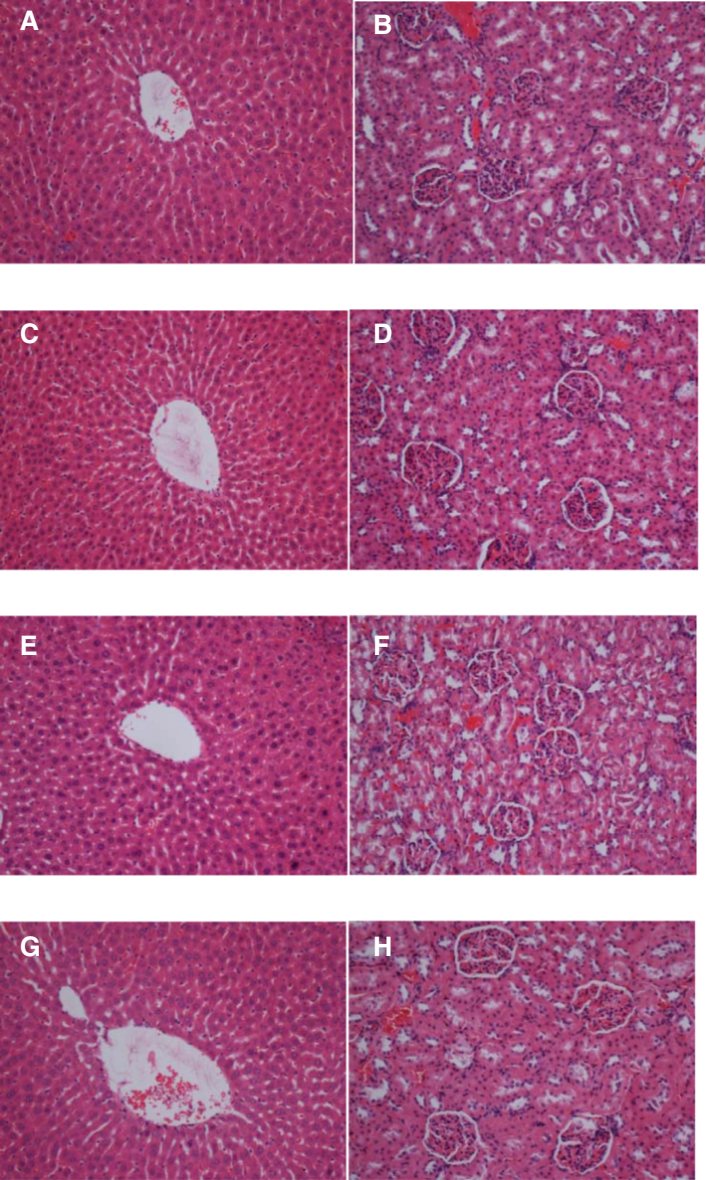
**Effects of HPTP on Histology of liver and kidney in acute toxicity testing.** Rats treated with vehicle (**1A** and **1B**), rats treated with 250 mg/kg HPTP (**1C** and **1D**), rats treated with 500 mg/kg HPTP (**1E** and **1F**), rats treated with 1000 mg/kg HPTP (**1G** and **1H**). There were no significant difference in the histology of liver and kidney between treated and control groups (H&E stain) (magnification 20x).

A single oral administration of (1-(4-hydroxy-phenyl)-3-m-tolyl-propenone) (HPTP) chalcone at three doses (250 mg/kg, 500 mg/kg or 1000 mg/kg) did not result any mortality for 24 hours and no toxic effects and abnormal behaviour (no sign of changes in eyes, fur, skin, respiration, sedation, convulsion) were observed throughout 14 days. The biochemical measurement of blood serum, clinical observation and histo-pathological estimation of the kidney and liver revealed that there are no significant differences (P > 0.05) between treated groups and normal control group as shown in (Tables 1, 2, 3), (Figure 1).

**Table 1:** **Effects of HPTP on renal function test (male and female rats) in acute toxicity studies**

**Male**	**Sodium mmol/L**	**Potassium mmol/L**	**Chloride mmol/L**	**CO**_**2**_ **mmol/L**	**An.gap mmol/L**	**Urea mmol/L**	**Creatinine μmol/L**
CMC 0.5%	147.8 ± 0.5	4.9 ± 0.2	112.8 ± 0.8	15.5 ± 0.78	24 ± 0.4	8.3 ± 0.6	31.5 ± 1.6
250 mg/kg	148.5 ± 2.2	4.8 ± 0.2	109.5 ± 2.8	15.6 ± 1.3	24.8 ± 0.9	6.2 ± 0.8	29.3 ± 1.6
500 mg/kg	149.5 ± 3.2	4.9 ± 0.4	111.5 ± 2.0	14.1 ± 0.5	24.8 ± 1.0	8.2 ± 1.3	30.0 ± 0.7
1000 g/kg	151.0 ± 2.4	5.2 ± 0.3	115.8 ± 1.7	13.8 ± 0.4	27 ± 1.9	11.3 ± 1.1	35.3 ± 2.3
**Female**							
CMC 0.5%	147.8 ± 0.6	4.9 ± 0.1	112.3 ± 1.7	13.4 ± 0.9	26.8 ± 0.5	7.2 ± 0.4	29.3 ± 1.3
250 mg/kg	147.8 ± 3.1	4.9 ± 0.1	109.3 ± 3.5	16.7 ± 1.2	27.3 ± 1.7	6.7 ± 1.1	30.0 ± 1.2
500 mg/kg	147.3 ± 1.6	5.3 ± 0.4	115.3 ± 3.7	14.3 ± 0.4	29.3 ± 1.0	7.8 ± 1.1	25.5 ± 2.0
1000 mg/kg	152.3 ± 1.7	5.4 ± 0.3	120.0 ± 2.5	14.0 ± 0.8	28.3 ± 3.0	6.6 ± 1.2	28.5 ± 2.3

Values are expressed as Mean ± SEM and One-way ANOVA was used for the analysis.

**Table 2:** **Effects of HPTP on liver functions in rats (male and female) in acute toxicity studies**

**Animals**	**T. protein (g/L)**	**Albumin (g/L)**	**Globulin (g/L)**	**T. billirubin (μmol/L)**	**ALP (IU/L)**	**ALT (IU/L)**	**AST (IU/L)**
**Male**							
CMC 0.5%	58.5 ± 2.50	14.0 ± 0.8	45.0 ± 2.0	3.3 ± 0.3	97.5 ± 3.6	55.8 ± 1.5	171.0 ± 5.5
250 mg/kg	53.0 ± 2.6	13.3 ± 1.4	47.5 ± 2.8	4.3 ± 0.5	87.0 ± 8.3	46.5 ± 2.2	183.5 ± 11.2
500 mg/kg	55.8 ± 0.9	14.8 ± 0.6	54.8 ± 2.7	3.3 ± 0.3	101.3 ± 8.1	56.3 ± 3.7	189.3 ± 6.8
1000 mg/kg	55.8 ± 1.1	14.8 ± 1.1	52.8 ± 3.1	4.0 ± 0.4	105.5 ± 6.7	59.3 ± 4.9	176.3 ± 14.5
**Female**							
CMC 0.5%	59.8 ± 1.8	13.0 ± 0.4	42.5 ± 0.9	3.5 ± 0.3	87.5 ± 1.9	55.3 ± 2.8	170.0 ± 4.3
250 mg/kg	58.5 ± 2.5	12.8 ± 1.0	48.0 ± 1.8	4.0 ± 0.4	85.0 ± 3.9	56.0 ± 3.9	189.5 ± 12.7
500 mg/kg	55.0 ± 2.1	15.3 ± 0.6	50.8 ± 3.7	3.8 ± 0.5	93.0 ± 6.4	54.8 ± 2.7	194.5 ± 11.7
1000 mg/kg	55.5 ± 2.3	15.3 ± 0.8	49.0 ± 2.8	3.8 ± 0.5	104.3 ± 4.3	60.0 ± 4.2	215.8 ± 13.0

Values are expressed as Mean ± SEM and One-way ANOVA was used for the analysis.

**Table 3:** **Effects of HPTP on lipid profile levels in blood serum of male and female rats in acute toxicity studies**

**Animals**	**Triglyceride**	**T. cholesterol**	**HDL**
**Male**			
CMC 0.5%	0.8 ± 0.1	2.1 ± 0.4	2.1 ± 0.1
250 mg/kg	0.7 ± 0.0	2.1 ± 0.2	2.9 ± 0.2
500 mg/kg	0.72 ± 0.1	2.3 ± 0.3	2.4 ± 0.2
1000 mg/kg	1.1 ± 0.1	2.0 ± 0.1	2.6 ± 0.1
**Female**			
CMC 0.5%	0.7 ± 0.1	1.9 ± 0.1	2.0 ± 0.3
250 mg/kg	0.7 ± 0.0	1.8 ± 0.2	1.9 ± 0.2
500 mg/kg	0.7 ± 0.1	1.7 ± 0.2	2.0 ± 0.3
1000 mg/kg	0.7 ± 0.1	1.9 ± 0.1	2.2 ± 0.3

Values are expressed as Mean ± SEM and One-way ANOVA was used for the analysis.

### Antiulcer characteristics

The gastro-protective effects of HPTP chalcone was estimated against indomethacin induced gastric ulcer in rats (Table 4) in terms of pH of gastric, mucus barrier, erosive gastric damage area and percentage of inhibition. Pre-treatment with 50 mg/kg and 100 mg/kg of HPTP were found to inhibit stomach lining injury induced by indomethacin. This inhibition appeared in low dose and high dose (65.4% and 74.4%) respectively. Thus, this study revealed that HPTP can decrease the acidity significantly (p < 0.05) which plays a major role in stimulating stomach ulcer. Gastric mucus layer that is covering the gastric lining plays an important role in preserving the inner layer of stomach from exogenous aggressive factors. In this study it has been shown that HPTP significantly enhanced the gastric mucus production at low dose and high dose administration (Table 4).

**Table 4:** **Effects of HPTP on gastric ulcer induced by indomethacin in rats**

**Pre-treatment (5 ml/kg)**	**Gastric pH**	**Mucus barrier μg/g tissue**	**Ulcer area (mm** ^**2**^ **)**	**% inhibition**
Normal (0.5% CMC)	6.36 ± 0.24*	27.67 ± 1.97*	----	----
Indomethacin 100 mg/kg	2.17 ± 0.07	7.17 ± 0.21	112.32 ± 11.74	----
HPTP 50 mg/kg	3.10 ± 0.18*	21.97 ± 1.32*	38.88 ± 2.88*	65.4
HPTP 100 mg/kg	4.52 ± 0.17*	27.48 ± 1.08*	28.80 ± 2.28*	74.4
Omeprazole 20 mg/kg	5.7 ± 0.30*	28.43 ± 1.45*	10.08 ± 1.76*	91.04

Values are expressed as Mean ± SEM. (*) indicates significant at *P* < 0.05 versus indomethacin (ulcerated) group with pre-treatment groups.

**Figure 2:**
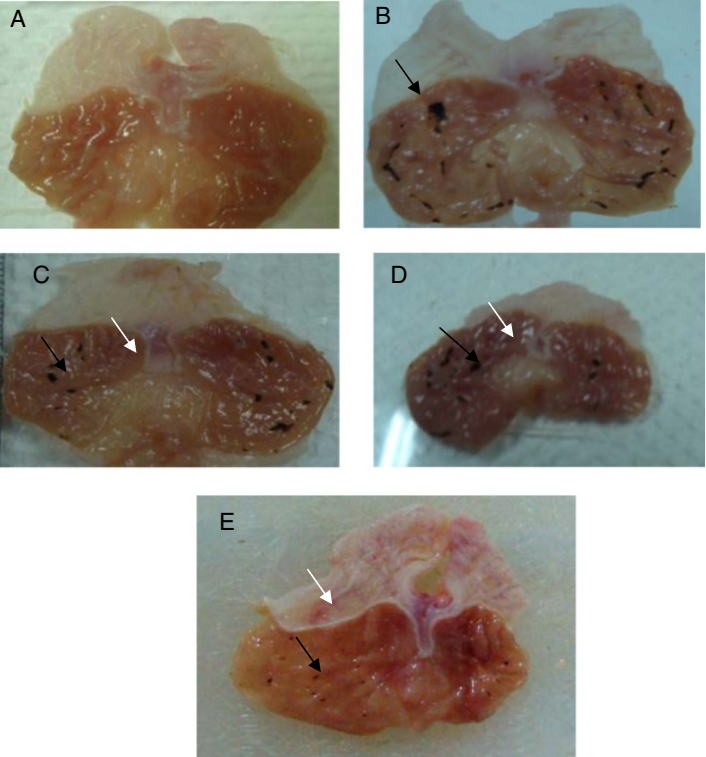
**Macroscopic evaluation of the gastric lesions in rats.** The normal control group **(A)** shows no injury of gastric mucosa. The indomethacin treated group **(B)** produced visible hemorrhagic necrosis of gastric mucosa (black arrow). Rats pre-treated with 50 mg/kg HPTP **(C)** shows moderate injuries in the gastric mucosa (black arrow) and little flattened (white arrow). Rats pretreated with 100 mg/kg HPTP **(D)** shows little flattened (white arrow) in gastric mucosa and fewer injuries were observed (black arrow). The omeprazole treated group **(E)** has more flattened (white arrow) of gastric mucosa and few and small injuries in the gastric mucosa (black arrow).

The macroscopic sections of stomachs showed significant differences between groups (Figure 2). Rats in the HPTP treated groups (3 and 4) significantly reduced areas of gastric lesions compared to rats in group 2. Indomethacin induced gastric lesions were significantly reduced in term of size and severity in rats pre-treated with omeprazole as shown in Figure 2.

### Antioxidant properties

**Figure 3:**
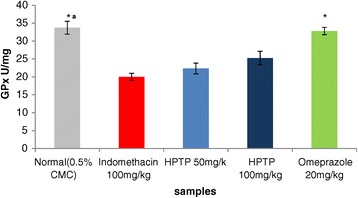
**Effects of HPTP on glutathione peroxidase activity in indomethacin induced erosive gastric damage in rat stomach.** Values are expressed as Mean ± S.E.M. (*^a^) indicates significance at *P < *0.05 versus indomethacin with HPTP chalcone and (*) at *P < *0.05 versus indomethacin with normal control.

**Figure 4:**
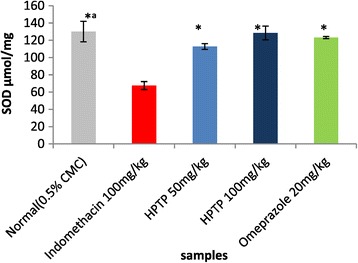
**Effect of HPTP on SOD enzyme in indomethacin induced erosive gastric damage in rat stomach.** Values are expressed as Mean ± SEM. (*) indicates significance at *P* < 0.05 versus indomethacin with HPTPchalcone and (*^a^) at *P < *0.05 versus indomethacin with normal control.

**Figure 5:**
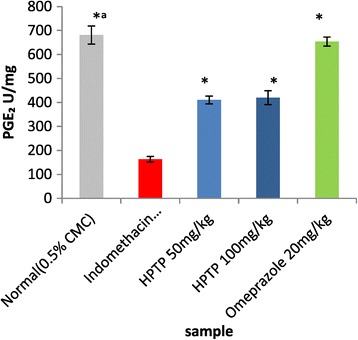
**Effect of HPTP on Prostaglandin E**_**2**_ **activity.** Values are expressed as Mean ± S.E.M. (*^a^) indicates significance at *P < *0.05 versus indomethacin with HPTP chalcone and (*) at *P < *0.05 versus indomethacin with normal control.

**Figure 6:**
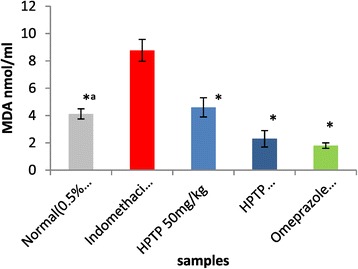
**Effect of pre-treatment with HPTP and omeprazole followed by indomethacin (100 mg/kg) on Malondialdehyde releasing.** Values are expressed as Mean ± S.E.M. (*^a^) indicates significance at *P < *0.05 versus indomethacin with normal control and (*) at *P* < 0.05 versus HPTP chalcone.

The enzymatic and non-enzymatic parameters that play important role in protecting the gastric mucosa from damage. This study evaluated the effect of ulcer induction on some of these parameters (GPx, SOD, PGE_2_ and MDA), thus the effect of HPTP pre-treatment on these parameters production. It has been found that in indomethacin group GPx, SOD, and PGE2 were significantly lower than in normal control (Figures 3, 4 and 5), while MDA was significantly higher in indomethacin group than in control (Figure 6). This indicated that indomethacin increase the level of MDA and decrease the level of SOD, GPX and PGE_2_. As for HPTP, this study revealed that chalcone significantly increased SOD and PGE2 at low and high dose, while HPTP chalcone increased GPx level but no significant differences between indomethacin group and HPTP groups. In this study it has been shown that MDA level in indomethacin group was increased but HPTP chalcone at low and high doses decreased MDA that play important role in gastric ulcerogenic effects (Table 5).

**Table 5:** **SOD, GPx, PGE**_**2**_ **and lipid peroxidation(MDA) properties of HPTP in indomethacin-induced gastric lesions in rats**

**Pre-treatment (5 ml/kg)**	**GPX (μmol/mg)**	**SOD (μmol/mg)**	**PGE2 (U/ml)**	**MDA (nmol/ml)**
Normal (0.5% CMC)	33.72 ± 1.80*^a^	130.06 ± 11.58*^a^	681.03 ± 37.83*^a^	4.12 ± 0.37*^a^
Indomethacin 100 mg/kg	20.02 ± 0.97	67.55 ± 4.63	163.07 ± 11.68	8.78 ± 0.78
HPTP 50 mg/kg	22.33 ± 1.47	112.78 ± 3.31*	410.52 ± 16.38*	4.6 ± 0.67*
HPTP 100 mg/kg	25.22 ± 1.93	128.42 ± 7.98*	419.85 ± 28.92*	2.25 ± 0. 59*
Omeprazole 20 mg/kg	33.23 ± 1.11*	125.16 ± 1.53*	653.77 ± 19.99*	1.79 ± 0.19*

Values are expressed as Mean ± SEM. (*^a^) indicates significance at *P* < 0.05 versus normal control with indomethacin group, (*) *P* < 0.05 versus indomethacin with all treated groups. GPx = Glutathione peroxidase, SOD = Superoxide dismutase, PGE2 = Prostaglandin E_2_, MDA = Estimation of lipid peroxidation level.

### Histological examination

**Figure 7:**
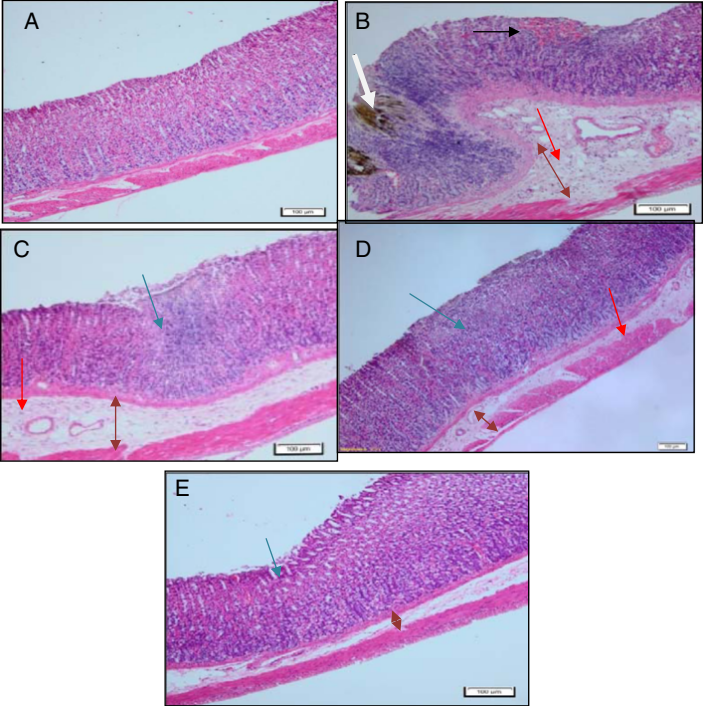
**Histological evaluation of gastric lesions in sections stained with Hematoxylin & Eosin (10X).** Normal control group **(A)** has normal tissue epithelium. Indomethacin group **(B)** shows disruption of surface epithelium with hemorrhage (black arrow) and the lesions infiltrate deep into mucosa layer (white arrow) wide edema (brown arrow) and leukocyte infiltration (red arrow). HPTP pre-treated groups (**C** & **D**) shows reduction of submucosal edema (brown arrow), mild to moderate disruption of mucosa epithelium (blue arrow) few infiltration of leukocyte. Omeprazole group **(E)** shows mild disruption of the surface epithelium (blue arrow), and mild submucosal edema (brown arrow) and bit of leukocyte (H&E stain, magnification 20x).

**Figure 8:**
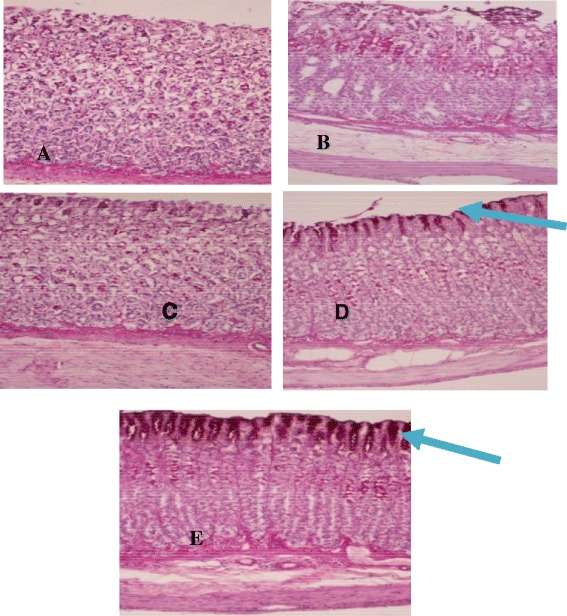
**Histological evaluation of gastric glycoproteins in sections stained with PAS.** The magenta color in the apical epithelial cells shows glycoprotein accumulation in the gastric glands (blue arrow). Normal control group **(A)**, indomethacin treated group **(B)**, HPTP pre-treated groups (**C** & **D**) and omeprazole pre-treated group **(E)** (PAS stain, magnification 20x).

Histological examinations of group 1 indicated that there was no disruption of the surface epithelium, while the histological examination showed extensive damage to the gastric mucosa in group 2, with necrotic lesions penetrating deeply into the mucosa accompanied by extensive edema and leukocyte infiltration of the submucosal layer (Figure 7). Group 3 exhibited moderate disruption of the surface epithelium, with edema and leukocyte infiltration of the submucosal layer, and group 4 showed a mild disruption of the surface epithelium with edema and leukocyte infiltration into the submucosal layer. Group 5 showed mild edema and leukocyte infiltration of the submucosal layer, but no disruption of the surface epithelium. These results demonstrated that the HPTP exerted cytoprotective effects in a dose-dependent manner (Figure 5).

(Figure 8) shows the Periodic Acid-Schiff (PAS) stain. The gastric mucosa in animals pretreated with HPTP or omeprazole (group 3–4) displayed increased PAS staining intensity compared to the rats in group 2, indicating an increase in the glycoprotein content of gastric mucosa in pretreated rats (Figure 8).

**Figure 9:**
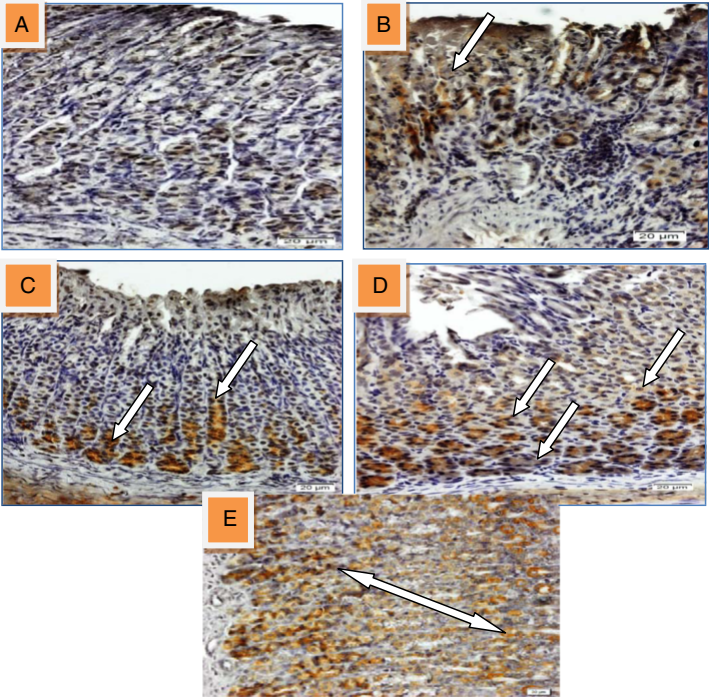
**Immunohistochmical analysis of HSP70 in gastric tissue induced with indomethacin. (A)** Section showed normal mucosal. **(B)** Section of ulcerated stomach tissue showed down-regulation of HSP70 in injured area (white arrow). Sections (**C**, **D** and **E**) that pre-treated with 50, 100 mg/kg of HPTP and 20 mg/kg of omeprazole respectively showed up-regulation of HSP70 (white arrow) (magnification 20x).

**Figure 10:**
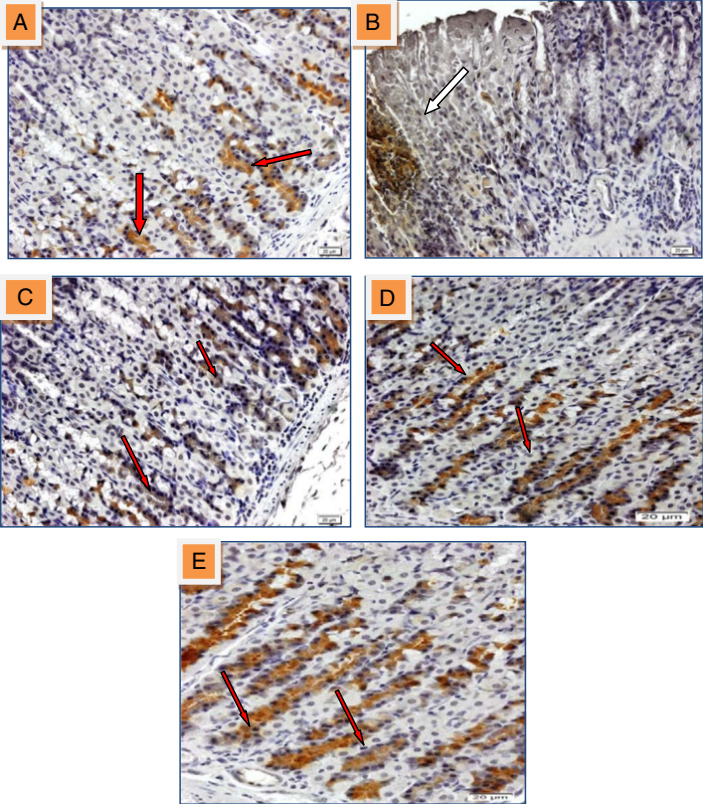
**Immunohistochmical analysis of TGF-β protein expression in gastric tissue of rat induced with indomethacin. (A)** Section shows normal mucosal area and express regulated of TGF-β (red arrow). **(B)** Section of ulcerated stomach tissue ashowed down-regulation of TGF-β almost non-existent in injured area (white arrow). Sections (**C**, **D** and **E**) that pre-treated with 50, 100 mg/kg of HPTP and 20 mg/kg of omeprazole respectively found the up-regulation of TGF-β (red arrows) (magnification 20x).

The expression of the HSP70 and TGFβ protein in the gastric mucosa was down-regulated in section of ulcerated stomach tissue while it was up regulated in the groups pre-treated with 50, 100 mg/kg of HPTP and 20 mg/kg of omeprazole respectively (Figures 9 and 10).

**Figure 11:**
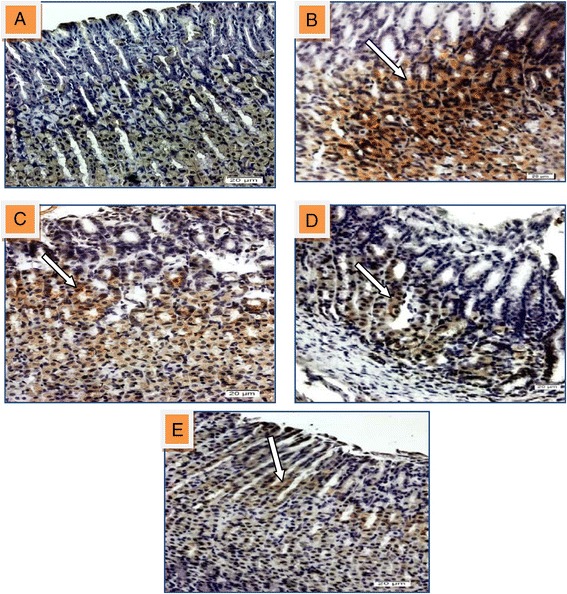
**Immunohistochmical analysis of Bax protein expression in gastric tissue of rat induced with indomethacin. (A)** Section shows normal mucosal area and express down-regulated of Bax. **(B)** Section of ulcerated stomach tissue appears up-regulation of Bax protein in injured area (white arrow). Sections (**C**, **D** and **E**) that pre-treated with 50, 100 mg/kg of HPTP and 20 mg/kg of omeprazole respectively found the Bax protein expression was down-regulated (magnification 20x).

Immunohistochmical analysis of Bax protein expression in gastric tissue of ulcerated stomach showed up regulation of Bax protein in injured area while it was down regulated in groups pre-treated with 50, 100 mg/kg of HPTP and 20 mg/kg of omeprazole respectively (Figure 11).

## Discussion

Chalcones are widely found in the plants and produce antioxidant, antimicrobial, anticancer and antibiotic properties. Their availability in different plant sources as well as their pharmacological values and low toxicity resulted in the interest of scientists and industries [17]. This study was performed with the aim of evaluating antiulcer effects of tested chalcone in rats. The results of this study revealed that all doses (250,500,1000 mg/kg) of (1-(4-hydroxy-phenyl)-3-m-tolyl-propenone) (HPTP) chalcone are safe because none of the animals produced toxic signs and symptoms and none died during the study period. Moreover there are no significant differences in biochemical parameters of liver and kidney. Histology revealed no nephrotoxic or hepatotoxic effect. Similarly, several researcher used various synthesized Schiff base compound and showed no nephrotoxicity or hepatotoxicity and the biochemical parameter are within the normal rang [7-9]. Based on these findings the doses of chalcone used for the antiulcer experiment was of no toxicity to rats. Moreover, this study showed that chalcone was able to produce more ulcer inhibition in gastric tissue in rats (65.4% in low dose and 74.4% in high dose administration of chalcone. Kyoguku et al. [18] conducted their study with the aim of comparing antiulcerogenic effects of different chalcones and therefore did not report on the protective mechanism of chalcone. This study revealed that this compound produces its dose dependent protective effect through the increase in the gastric wall mucus and as well as increasing the PH and antioxidant properties of mucosa. With the consistence of the result of our study, previous studies also showed anti ulcerogenic effects of various synthesized compound against ethanol-induced gastric ulcerogenic damage and showed increased in gastric wall mucus [19]. In terms of antioxidant properties, HPTP chalcone significantly showed to be capable of reducing lipid peroxidation as well as improving SOD and PGE_2_ and GPX levels. In this study significant difference was observed in terms of SOD and PGE2 levels between the experimental groups. It was previously reported that the administration of indomethacin at doses as low as 25 mg/kg resulted in significant decrease in GPx levels in rats [20,21]. The higher doses of indomethacin were used in this study and significant reduction in GPx was observed in this group compared with the normal controls and treated groups. These findings confirm the antiulcer protection of chalcone based on prevention or reduction of oxidative stress through increase in production of SOD and PGE_2_ and GPx levels. It has been revealed that gastric hyperacidity and ulceration of the stomach mucosa can be to be mediated largely through the generation of excessive reactive oxygen species and free radicals, especially the hydroxyl radical which leads to oxidative stress [22]. It is proven that antioxidants have anti-ulcerogenic properties through neutralization of free radicals and breakdown the reactive oxygen species (ROS) chain process [23]. Odabasoglu et al. (2006) also reported that indomethacin administration resulted in significant reduction in SOD levels in rats. This was in line with findings of this study that revealed a significant decrease in SOD levels in rats with indomethacin induced gastric ulcer. This study also revealed that administration of chalcone is capable of increasing the SOD levels and therefore play a preventive role against indomethacin-induced gastric ulcerogenic damage. Koizumi et al. [24] showed that the administration of 30 mg/kg indomethacin resulted in a significant reduction in the PGE_2_ levels in rats. This finding was also in line with the findings of this study. This study revealed that the most important oxidative mechanisms of indomethacin induced erosive gastric damage is related to reduction of the important antioxidants in the stomach including SOD as well as PGE_2_. In the present study, the findings from histology tests showed the multiple potential effect of chalcone through reduction in acidity and inhibition percentage, while in the same time mucus barrier was increased, which resulted in smaller gastric ulcer area in chalcone groups (Table 4). These properties were also found to be dose dependent as they improved gastric mucosal state by increasing dosage. Furthermore, this study revealed that administration of chalcone at low and high dose (50 &100 mg/kg body weight) reduced ulcers in stomach. These findings were in line with the previous findings in terms of protective mechanisms of chalcone and flavonoids [25-27]. The findings of this study suggest the use of chalcone with NSAIDs to reduce the risk of PUD. Also in a study by Okunrobo and colleagues [28] they tested the anti-inflammatory and gastro-protective properties of a synthetic chalcone with different dosages on rats who compared with the acetylsalicylic acid (ulcer control) and cimetidine (reference) rat groups. The results were similar with the results present study terms of gastric wall mucosal characteristics (ulcer area size, barrier and inhibition of gastric wall mucosa) and acidity of gastric content. Finally omeprazole was used as antiulcer reference chemicals in this study against induced ulcer by indomethacin. Omeprazole as a proton pump inhibitors (PPIs) are broadly prescribed medications with the control of gastric acid secretion for the prevention or treatment of peptic ulcer [29]. Omeprazole can suppress the gastric acid secretion through the H+,K + −ATPase [30]. It has been discovered that the H+,K + −ATPase as gastric cell proton pump transfers H+ in exchange for luminal K+ to generate a highly acidic environment in the stomach that is responsible for gastric acid secretion [31]. Lastly indomethacin as a potent inhibitor of prostaglandins synthesis diminishes the protective effect of PGE_2_ on mucosa of gastric wall [22].

## Conclusion

Acute toxicity tests did not show any sign of morbidity or mortality up to 1000 mg/kg of HPTP. Rats pre-treated with 50 mg/kg or 100 mg/kg of HPTP before administration of 100 mg/kg of indomethacin significantly protect erosive gastric mucosal damage compared to ulcerogenic control group. Histologic findings showed that HPTP remarkably protect gastric mucosa compared to ulcerogenic control group. The gastroprotective effects of HPTP could be attributed to increase in pH and mucus content of gastric content, increase endogenous enzymes and PGE2, and decrease in MDA level in gastric homogenate, over-expresion of HSP70 and TGF-β proteins, and down-expression of Bax protein.

## Methods

This experiment was approved by the animal ethic committee of animal experimentation, Faculty of Medicine, UM, ethic NO.PM/29/06/2012/SMD (R).

### Animals

Adult healthy *Sprague Dawley* (SD) rats were used for gastro-protective and (ICR) mice for acute toxicity evaluation. The animals were supplied from the animal house, Faculty of Medicine, UM, Kuala Lumpur. All experiments were designed to use the minimum number associated with valid statistical analysis. Study animals were housed at least 3 days before experiment under standard condition (individual plastic cages with wide mesh bottom for preventing coprophagia and temperature of 24 ± 2°C and lighting). Animals were fed with commercial diet and tap water [32]. The experimental animals were sacrificed after being anesthetized by 0.1 ml/100 gram body weight of a mixture of ketamine (8.75 ml, 100 mg/ml) and xylazine ( 1.25 ml, 100 mg/ml) [33,34].

### Chemicals

(1-(4-hydroxy-phenyl)-3-m-tolyl-propenone (HPTP) chalcone was obtained from Pharmacy Department, Faculty of Medicine, UM. HPTP was suspended in 5 ml/kg body weight of 0.5% w/v of Carboxylmethylcellulose (CMC) 30 minutes prior to the experiment. Indomethacin was purchased from sigma chemicals and used as ulcerogenic agent to induce gastric ulcers. Omeprazole was used as standard ulcer preventing agent, it was provided from University Malaya Medical Centre. Experimental chemicals were suspended in 0.5% carboxyl methyl cellulose (CMC) 30 minutes before feeding the rats and were administered orally by orogastric tube. All chemicals for laboratory experimentation were purchased from sigma chemical. Laboratory kits for prostaglandin E2 (PGE2), Glutathione peroxidase (GPX), superoxide dismutase (SOD) and Malondialdehyde (MDA) were purchased from Cayman chemicals. Blood samples were analysed in the Clinical Diagnostic Laboratory (CDL), University Malaya Medical Centre (UMMC).

### Acute toxicity

This test was performed to identify the safe dose for HPTP based on the OECD 423 guideline. HPTP was administered at 250, 500 and 1000 mg/kg body weight. All mice were deprived from food for 24 hours and water was withdrawn from the mice 2 hour before commencement of the experiment. Forty mice were equally divided in to four groups 5 male and female mice each:

- Group 1 (normal control) received vehicle (5 ml/kg CMC)
- Group 2 received (a dosage of 250 mg/kg) HPTP chalcone
- Group 3 received 500 mg/kg HPTP chalcone
- Group 4 received 1000 mg/kg HPTP chalcone

A single dose of the compound was administered to mice. Following administration , the animals were observed for abnormal behaviour (sedation, convulsions, respiratory distress, salivation, changes in skin, fur and etc.) for first 3 hours continuously and at 24 hours for mortality and at least once daily thereafter for 14 days. Feeding was replaced approximately 3–4 hours after dosing [9]. The animals were sacrificed on the 15^th^ day; blood sample was collected for biochemical assay including liver and kidney function. Liver and kidneys were preserved in buffered formalin 10% for 24 hours for histological study.

### Gastro-protective activity

In this study, indomethacin as a non-steroidal anti-inflammatory drug (NSAID) was used as ulcerogenic agent [35]. A total of 30 adult female SD rats weighting (200–250 gram) were assigned to 5 groups. All groups were fasted for 24 hours prior to the experiment, but allowed free access to drinking water until 2 hours before beginning the experiment. Group 1 and 2 were received CMC. HPTP at either dose of (50, 100 mg/kg body weight) was administered orally to groups 3 and 4, respectively. Omeprazole was administered to group 5 at a dose of 20 mg/kg. After 30 minutes all groups, except group 1, received indomethacin (100 mg/kg Six hours later, the animals were anesthetized by ketamine and xylazine then sacrificed by cervical dislocation [36]. The stomachs were excised quickly after tying the pyloric and cardiac ends and were kept in normal saline. The protective effects of HPTP were, as well, compared with gastric ulcerogenic model observed in rats that were treated with PPI (omeprazole).

### Measurement of gastric acidity:

The gastric juice was collected and centrifuged at 4000 rpm for 10 minutes in room temperature. The supernatant was used to measure the acidity of gastric juice by titration with 0.1 N NaOH solution using digital PH meter and the acid content was measured in mEq/L [7].

### Evaluation of macroscopic gastric mucosal lesions

The stomach was opened out along the greater curvature and washed slightly with normal saline. The erosive gastric damage formed on the inner surface of the stomach were counted under a dissecting microscope (1.8X) with square –grid eye piece to assess the ulcers. Ulcer area was then calculated based on the formula proposed by Salga et al. [37]. The lesion area was measured as (lesions area = lesion number x 1.8 x4).

$$ \left(\%\right)\ \mathrm{Inhibition} = \mathrm{Mean}\ \mathrm{ulcer}\ \mathrm{area}\ \left(\mathrm{control}\right) - \mathrm{Mean}\ \mathrm{treatment}/\mathrm{mean}\ \mathrm{ulcer}\ \mathrm{area}\ \left(\mathrm{control}\right) \times 100. $$

### Mucus barrier estimation

Gastric wall mucus was determined based on the modified procedure of Corne et al. [38]. The glandular portion of the stomach was immersed in 10 ml of 0.1% alcian blue solution which contains 1 g/L alcian blue, 0.16 M/L sucrose and sodium acetate 0.05 M/L, PH adjusted to 5.6 with HCL for 2 hours. The sample was then washed by 0.25 M/L sucrose for 15 minutes and then washed for 45 minutes to remove unbounded dye. Magnesium chloride (MgCl_2_) (10 ml of 0.5 M/L) was used to elute bound dye. For this purpose the sample was immersed in MgCl_2_ for 2 hours. The resulting blue solution was mixed and shaken with equal volume of diethyl ether for 2 min. The resulting emulsion was then centrifuged at 3000 rpm for 10 min. at room temperature. The absorbance of the aqueous layer was recorded at 605 nm using spectrophotometer. The standard curve was used to calculate the quantity of the extracted alcian blue per gram of glandular tissue (as μg alcian blue/gram tissue weight).

### Biochemical investigation of stomach tissues

Tissue homogenate was prepared by homogenizing 0.5 gram of gastric tissue on ice with 5 ml of phosphate buffered saline (PBS) using an ultra- turrax homogenizer for 15 minutes. The mixture was then centrifuged using refrigerated centrifuge at 4°C for 20 minute. The supernatants were collected in eppendorf tubes and used for enzymatic and non enzymatic assessments of the stomach tissue. SOD, GPX and MDA were assessed using the Cayman’s kits (Cayman chemical, USA) according to the manufacture’s protocols. While for measurement of Prostaglandin E2 (PGE2) stomach mucosal tissue (100 mg) was homogenized in1 ml homogenization buffer, which includes 0.1 phosphate buffer, PH7.4, 1 mM EDTA and 10 μM indomethacin. The sample was then centrifuged at 8000 rpm for 10 minutes to eliminate the particular matter. The supernatant was transferred to a clean eppendorf tube to evaluate PGE_2_ using prostaglandin E_2_ enzyme immune assay kit (Cayman Chemical Co.).

### Histological examinations

Stomach tissue samples were fixed in 10% buffered formalin for 48 hours and were then dehydrated by washing by ascending grades of ethanol. Samples were then cleared with xyline and embedded in paraffin wax. Then 5 μm thick slides were sectioned and stained with haematoxylin and eosin using conventional method and examined by light microscope. Following the method of McManus & Mowry [39] the glandular portion of the rat stomach sections were stained with Periodic Acid-Schiff (PAS) to observe the changes of glycoprotein.

### Immunohistochemistry

The immunohistochemical staining was conducted according to manufacturer’s protocol (Dako Cytomation, USA) to detect immunohistochemical antibodies such as HSP70, Bax and TGF-β. The tissue sections were place on Poly-L-lysine coated slides, then kept in oven at 60 (C°) for 24 hours in order to increase section adherence to the slide. The slides were deparaffinized by xylene and rehydrated by graded concentrations of alcohol, then placed in retrieval solution and incubated in microwave at high power for 15 min., the slides were cooled then washed with wash buffer. After that the tissue was covered with the (HSP70 1:1000), (TGF 1: 1000), and (Bax 1:50) biotinylated primary antibody and incubated for 30 minutes in dark and humid place, followed by rinsing the slides gently with wash buffer. Subsequently the tissue sections were covered with streptovidin peroxidase and incubated for 30 minutes, then rinsed gently by wash buffer and placed in humid and dark box. After wards the slides were covered with Diaminobenzidine (DAB) chromogen solution and incubated for10 minutes followed by washing with wash buffer, then by tap water. The next step is counter staining with Hematoxylin for 5 minutes followed by washing with tap water until the blue colour disappeared. Finally the tissue sections were dehydrated by graded alcohol, cleared by xylene and mounted as mentioned in the last steps in H&E staining method. The positive result was appeared brown in color.

### Statistical analysis

Descriptive analysis was performed for continuous variables and these variables were shown as mean ± standard error of measurement (SEM). One-way analysis of variance (ANOVA) was used to analyse the study variables between experimental groups in normally distributed data. P value less than 0.05 was considered as statistically significant at the confidence level of 95%.
